# Are Live Ultrasound Models Replaceable? Traditional versus Simulated Education Module for FAST Exam

**DOI:** 10.5811/westjem.2015.9.27276

**Published:** 2015-10-22

**Authors:** Suzanne Bentley, Gurpreet Mudan, Christopher Strother, Nelson Wong

**Affiliations:** * Icahn School of Medicine at Mount Sinai, Department of Emergency Medicine, New York City, New York; † Elmhurst Hospital Center, Department of Emergency Medicine, Elmhurst, New York; ‡ Massachusetts General Hospital, Department of Emergency Medicine, Boston, Massachusetts

## Abstract

**Introduction:**

The focused assessment with sonography for trauma (FAST) is a commonly used and life-saving tool in the initial assessment of trauma patients. The recommended emergency medicine (EM) curriculum includes ultrasound and studies show the additional utility of ultrasound training for medical students. EM clerkships vary and often do not contain formal ultrasound instruction. Time constraints for facilitating lectures and hands-on learning of ultrasound are challenging. Limitations on didactics call for development and inclusion of novel educational strategies, such as simulation. The objective of this study was to compare the test, survey, and performance of ultrasound between medical students trained on an ultrasound simulator versus those trained via traditional, hands-on patient format.

**Methods:**

This was a prospective, blinded, controlled educational study focused on EM clerkship medical students. After all received a standardized lecture with pictorial demonstration of image acquisition, students were randomized into two groups: control group receiving traditional training method via practice on a human model and intervention group training via practice on an ultrasound simulator. Participants were tested and surveyed on indications and interpretation of FAST and training and confidence with image interpretation and acquisition before and after this educational activity. Evaluation of FAST skills was performed on a human model to emulate patient care and practical skills were scored via objective structured clinical examination (OSCE) with critical action checklist.

**Results:**

There was no significant difference between control group (N=54) and intervention group (N=39) on pretest scores, prior ultrasound training/education, or ultrasound comfort level in general or on FAST. All students (N=93) showed significant improvement from pre- to post-test scores and significant improvement in comfort level using ultrasound in general and on FAST (p<0.001). There was no significant difference between groups on OSCE scores of FAST on a live model. Overall, no differences were demonstrated between groups trained on human models versus simulator.

**Discussion:**

There was no difference between groups in knowledge based ultrasound test scores, survey of comfort levels with ultrasound, and students’ abilities to perform and interpret FAST on human models.

**Conclusion:**

These findings suggest that an ultrasound simulator is a suitable alternative method for ultrasound education. Additional uses of ultrasound simulation should be explored in the future.

## INTRODUCTION

Ultrasound training is an essential part of many residency programs including emergency medicine (EM), obstetrics and gynecology, surgery and internal medicine.^1^–^3^ It is often included in the medical school clerkships within these fields. However, despite the fact that it has increasingly become a required skill for these specialties, ultrasound training varies greatly across training environments, programs, and specialties without standardized curriculums or assessment of skills^1^. For example, with the institution of the Accreditation Council of Graduate Medical Education Milestone project into EM residencies, expectations for incoming medical students have been clearly delineated.^4^ A survey of EM interns reported the largest gap in training between the undergraduate curriculum and the current competency-based expectations lies in the ultrasound milestone. Only 61% of responders felt they had received the equivalent of Level 1 training in ultrasound (as opposed to 99% in professional values and team management.^5^

The expansion of the clinical indications for ultrasound highlights the potential impact of innovative ultrasound education methods. The focused assessment with sonography for trauma (FAST) is an essential scan and was chosen as the focus for this study due to its potential application across fields, including EM, surgery and obstetrics. Traditional teaching in ultrasound is often expensive and time-consuming, requiring the use of live human models, instructors and ultrasound machines. Even with intensive resource utilization, traditional models have been shown to lag behind in the development of US interpretation skills.^6^ The use of simulation for medical teaching has been shown to be feasible and useful in many different educational scenarios. A recent joint Council of Residency Directors and Academy of Emergency Ultrasound consensus document suggests simulators are a viable alternative for ultrasound training.^7^ Similar to use of human simulators, the use of ultrasound simulators has been shown to be high fidelity and have the ability to enhance learning and evaluation. Simulators provide the experience of conducting an ultrasound by requiring proper probe placement and scanning techniques, and providing real-time ultrasound images as feedback. Several studies have been done validating simulators for ultrasound-guided procedures including central line placement and paracentesis.^8^–^10^ A prior study looked at the use of a simulator in the teaching of the FAST to acquire and interpret images and showed no significant difference between the live model and ultrasound simulator groups.^11^

This study sought to evaluate the question: “are live ultrasound models replaceable?” through a more thorough education strategy and assessment requiring image acquisition and interpretation, in addition to assessment on validated American College of Emergency Physician (ACEP) ultrasound questions and clinical indications and applications of FAST use. The aim of this study was to show non-inferiority of a simulator-based ultrasound training module compared to the traditional model using human models, which is a more expensive and more time-consuming educational paradigm. For this study, it was hypothesized that medical student use of an educational module for ultrasound education using a sonographic simulator during their fourth-year EM clerkship would not be inferior to traditional teaching using live lecture followed by hands-on training with live models.

## METHODS

### Study Design

This was a prospective, blinded, controlled study conducted on a consecutive sample of medical students participating in a fourth-year EM clerkship. This study was approved by the institutional review board, participation was voluntary, and verbal consent was obtained from all participants.

### Study Setting and Population

This study was conducted over eight months, consecutively enrolling medical students during their one month, required fourth-year medical student EM clerkship at an urban, academic, tertiary care medical center and its affiliates. No formal ultrasound education exists in the medical student curriculum at this institution. Students were randomized into a traditional training group (control group) or an ultrasound simulator group (intervention group). Randomization was based on months of the year with students rotating during odd numbered months assigned to the control group and students rotating during even numbered months assigned to the intervention group.

### Study Protocol

The training was performed once a month for each group of students. Two weeks prior to the training, all students took a 20-question written pretest, composed of questions from the ACEP ultrasound question bank, a validated question bank targeting emergency department ultrasound indications, and ultrasound image interpretation. Along with the pre-test, students took a survey evaluating their baseline knowledge, prior exposure to and comfort level with ultrasound. The survey questions were based on a Likert scale of 1 through 4 with 1 representing “not at all” comfortable to 4 representing “very comfortable” with the item.

All students received a standardized, introductory lecture on the use of ultrasound, FAST basics and indications, and how to conduct a FAST exam. No student questions were answered during the lecture to maintain standardization. The lecture was delivered by the same instructor to both control and intervention groups, prior to opportunity for hands-on, self-directed practice. Noble et al demonstrated that practical training was an important part of ultrasound education.^12^ The instructor demonstrated image acquisition for each of the two groups and then participants were given time for self-directed, non-proctored practice on model of their assigned group. The control group participated in hands-on learning and practiced the FAST exam on human models (student volunteers). The intervention group participated in hands-on learning and practice of the FAST exam on the ultrasound simulator, a SonoMan Ultrasound Diagnostic Trainer (Simulab, Seattle, WA), which is a torso model with embedded electronics that simulates high fidelity normal and pathologic images in real time as the students perform ultrasound scans. No pathologic images were included or accessed during this training module.

Following the training modules, all students completed a post-test (identical to the pre-test), as well as a repeat survey. Additionally, an objective structured clinical examination (OSCE) on a live model was administered to both groups. The live model was used for the examination because the ultimate goal is to improve the ability to perform ultrasound on a live patient in a clinical setting. Students were assessed via OSCE on their performance of the FAST exam using a standardized, clinical skills “Critical Action” checklist (Figure) administered and graded by two blinded facilitators, both expert in emergency ultrasound. Examples of items on the checklist include proper probe orientation and the ability to effectively visualize each ultrasound view of the FAST exam.

### Outcome Measurements

Outcomes based on the following measures were evaluated: comparison of ultrasound knowledge between pre- and post-test scores in order to assess ultrasound knowledge; comparison of pre- and post-survey results of comfort with use of ultrasound; and finally the results of the OSCEs, specifically the ability of a student to perform critical actions required in order to successfully identify and interpret normal and pathologic images on FAST. We analyzed knowledge and comfort within groups from pre to post intervention, as well as between groups. OSCE scores of ability to perform ultrasounds were compared between groups.

## RESULTS

All clerkship students offered participation consented and a total of 93 students were trained and tested in this study (control group N=54, intervention group N=39). There was no significant difference between groups on pre-test scores, survey results of prior ultrasound training and education or comfort level using ultrasound in general and specifically for the FAST exam. All students were in their fourth year of medical school, had similar levels of prior training in ultrasound, and similar initial comfort levels with ultrasound.

All students showed a significant improvement in their pre- and post-test scores (p<0.001). Mean pre-test and post-test scores for the control group were 58.5% (SD12) and 78.1% (SD 13), respectively. Mean pre-test and post-test scores for the intervention group were 56.7 % (SD13) and 75.4% (SD12). Comparison of scores between groups showed no significant difference.

Mean pre-survey comfort level was 1.38 on a four-point Likert score for the control group and 1.1 for the intervention group (p=0.81). Post-survey comfort level was 2.65 for the control group and 2.67 for the intervention group. All students in both control and intervention groups demonstrated significant improvement in their comfort levels using ultrasound in general and for the FAST exam after they received the intervention (p<0.001), with no difference between the two groups. All students reported scores of 3 or 4 on usefulness of educational session, again with no difference between groups. Additionally, there was no significant difference between groups on the OSCE standardized, clinical skills checklist conducted on the human model. Mean OSCE score was 78.2% for the control group and 81.6% for the intervention group. Overall, no difference in any of the described metrics was demonstrated between groups trained on human models versus those trained on the ultrasound simulator.

## DISCUSSION

It was hypothesized that using an ultrasound simulator would not be inferior to a human model for basic ultrasound training for the FAST exam. All students showed an increase in ultrasound knowledge, comfort and confidence after the educational intervention. In addition, the intervention group exhibited similar scores and comfort and confidence levels compared to the control group on the written, knowledge-based test and OSCE scores, which represents similar knowledge and skills gains.

Traditional ultrasound education using human models, direct faculty time, and a dedicated ultrasound machine is expensive and time consuming. Additionally, a downside to use of human models is the scarcity of pathologic examination findings. The use of an ultrasound simulator streamlines the educational process by obviating the need for human models and additional ultrasound machines for training purposes. Another notable advantage is that various pathology that would be impossible to recreate in healthy models can be demonstrated. ACEP advocates that trainees be exposed to both normal and pathologic examinations in order to increase proficiency and skill level.^13^

## LIMITATIONS

Limitations to the study include a small sample size (N=93). Data was only collected at one clinical center. Outcome measures chosen demonstrate knowledge acquisition but do not offer data on clinical or patient care outcomes. Additionally, we did not assess demonstration to date of long-term retention.

## CONCLUSION

The use of an ultrasound simulator is a convenient and objective method of educating medical students on ultrasound. Study results reveal that the use of a novel curriculum incorporating ultrasound simulation was non-inferior to traditional methods of ultrasound education using human models as demonstrated through knowledge-based written testing, surveys of comfort levels with ultrasound, and objective examinations of students’ abilities to perform ultrasound on a human model in real time. There is a paucity of literature on the subject of validated teaching and evaluation of bedside ultrasound. This study is a proposed step on the path to developing an ultrasound curriculum using simulation methods that are non-inferior to traditional methods for teaching ultrasound. The ultimate goal is to develop an exportable and easy-to-use module for self-directed ultrasound training that will eliminate the need for models, live instructors, and that may be used across many different specialties, levels of training, and practice settings. Ultrasound simulation provides a viable solution to the problem of deliberate practice and mastery of the FAST and other ultrasound applications.

Additional uses of ultrasound simulation should be explored in the future, in particular, perhaps pioneering a validated and standardized learner-directed module for ultrasound training.

## Figures and Tables

**Figure:**
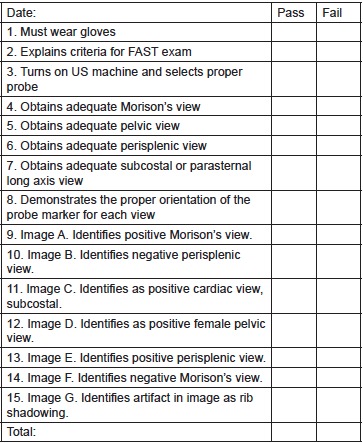
Critical action checklist for objective structured clinical examination of focused assessment with sonography for trauma (FAST) performance. *US*, ultrasound
